# EXPLICIT: a feasibility study of remote expert elicitation in health technology assessment

**DOI:** 10.1186/s12911-017-0527-0

**Published:** 2017-09-04

**Authors:** Bogdan Grigore, Jaime Peters, Christopher Hyde, Ken Stein

**Affiliations:** Evidence Synthesis & Modelling for Health Improvement (ESMI), Institute for Health Research, University of Exeter Medical School, University of Exeter, Room 3.09.3, St Luke’s Campus, Heavitree Road, Exeter, EX1 2LU UK

**Keywords:** Expert elicitation, Probability distributions, Elicitation tool, Email elicitation, Probabilistic decision modelling

## Abstract

**Background:**

Expert opinion is often sought to complement available information needed to inform model-based economic evaluations in health technology assessments. In this context, we define expert elicitation as the process of encoding expert opinion on a quantity of interest, together with associated uncertainty, as a probability distribution. When availability for face-to-face expert elicitation with a facilitator is limited, elicitation can be conducted remotely, overcoming challenges of finding an appropriate time to meet the expert and allowing access to experts situated too far away for practical face-to-face sessions. However, distance elicitation is associated with reduced response rates and limited assistance for the expert during the elicitation session. The aim of this study was to inform the development of a remote elicitation tool by exploring the influence of mode of elicitation on elicited beliefs.

**Methods:**

An Excel-based tool (EXPLICIT) was developed to assist the elicitation session, including the preparation of the expert and recording of their responses.

General practitioners (GPs) were invited to provide expert opinion about population alcohol consumption behaviours. They were randomised to complete the elicitation by either a face-to-face meeting or email. EXPLICIT was used in the elicitation sessions for both arms.

**Results:**

Fifteen GPs completed the elicitation session. Those conducted by email were longer than the face-to-face sessions (13 min 30 s vs 10 min 26 s, *p* = 0.1) and the email-elicited estimates contained less uncertainty. However, the resulting aggregated distributions were comparable.

**Conclusions:**

EXPLICIT was useful in both facilitating the elicitation task and in obtaining expert opinion from experts via email. The findings support the opinion that remote, self-administered elicitation is a viable approach within the constraints of HTA to inform policy making, although poor response rates may be observed and additional time for individual sessions may be required.

## Background

Expert opinion is often sought to complement available information needed to inform model-based economic evaluations in health technology assessments (HTAs). In many cases, experts are invited to provide quantitative judgements on unknown model parameters [1]. In the context of HTA, we define expert elicitation as the process of a subject expert specifying a quantity of interest and associated uncertainty around it, which can be encoded as a probability distribution [2]. This enables the probabilistic handling of quantities to address parameter uncertainty.

Expert elicitation is, however, not without risks: not only is guidance scarce for its use in HTA [3], contributing to increased methodological anxiety, but also an impressive body of literature exists on the biased nature of human judgement of probability. Possible common biases may relate to the training or experience of the expert, or even their current mood [4]. Motivational biases may include group thinking (when the desire for harmony or conformity in the group results in an irrational outcome), misinterpretation, wishful thinking, impression management (experts displaying high confidence to protect their public image despite having little actual knowledge on the topic) [5], and experts with the best exposure to the relevant topic wanting to formulate opinions to influence the outcome, irrespective of their true belief [6].

The dominant theory in the elicitation of probabilities is that of *cognitive biases*. In the 1970s, Tversky and Kahnemann [7] initiated a biases and heuristics research programme, which explored probability judgements. In their early work, they concluded that the instinctive strategies employed by humans to make probability judgements, referred to as heuristics, lead to systematic and predictable error. They concluded that humans were unlikely to achieve acceptable performance in probability elicitation. Their work was widely cited, discussed and adopted by many disciplines.

However, research interest is now slowly moving beyond the “heuristics and biases” research programme [8]. Evidence suggests that although the heuristics described by Tversky and Kahnemann are reproducible, the bias introduced by such strategies could be drastically reduced by changing the task characteristics [9–11]. Furthermore, in a study where experts provided estimates to technical questions in both their area of expertise and in areas in which they were not experts, Mullin found that, as experts, participants “were far more serious and cautious about the tasks than as non-experts” [12]. Finally, an accepted way to increase the robustness of elicited estimates against bias is to combine the opinions of multiple experts [13–16].

Even if the risk of bias associated with probabilistic judgement can be significantly reduced, elicitation amplifies the complexity by requiring multiple summaries from the expert to specify a single distribution [2], increasing the burden of the task. Given the enormous potential for a facilitator to reduce bias in expert elicitation, typical recommendations on conducting expert elicitation support a degree of face-to-face interaction with the expert during the elicitation session [2]. The role of the facilitator is to make sure that the questions are clear to the expert, that the expert’s belief is accurately recorded, and the facilitator may even point out fallacies in the expert’s judgement and allow the expert to revise their opinion. Furthermore, experts may feel more responsibility to complete a session with a facilitator than to complete an anonymous questionnaire [17].

Conducting a face-to-face elicitation requires a significant commitment of resources [17] (trained facilitator, travel costs, time); especially when multiple experts are involved. It can be very time consuming, especially in disease areas where there are few experts, who are geographically dispersed, with busy schedules. Moreover, during face-to-face elicitation, the facilitator needs to be careful not to impose their beliefs, or biases, onto the expert’s belief.

Previous work [18] highlighted the critical impact that expert availability has on elicitation, where low availability for face-to-face sessions led to recruitment taking a very long time. Besides ensuring that the question and task are clear to the expert; and the expert’s response is appropriately encoded to reflect their belief, expert elicitation in the HTA context usually has relatively short timeframes, especially for those HTAs informing national and local decision-making. Thus the time available for face-to-face meetings may be limited and might prevent getting estimates from certain experts.

The use of self-administered internet or email-based (distance) elicitation provides an opportunity to reduce time necessary for scheduling sessions, and reduce costs associated with travelling for the sessions, while reaching experts who are farther away. The expeditious nature of HTAs makes distance elicitation a very attractive solution, and examples of its use in HTA are available [19–22]. It also ensures that all experts receive exactly the same information on which to base their judgements and there is no bias introduced by a facilitator. The downside is the difficulty of formulating the question in a way that experts understand (without additional prompts), and a likely low response rate as with any survey (due to lack of motivation to respond or the cognitive burden of the exercise). However, in a recent ecological study that used self-administered elicitation together with facilitated elicitation, Baker et al. [23] found little difference between expert estimates that could be explained conclusively by the mode of administration.

Essentially, with distance elicitation, more experts can be reached in a shorter time, however usually at the cost of very low response rates, particularly when the target respondents are clinicians [24].

For any elicitation, it is expected that all experts would have the required substantive expertise (knowledge on the relevant topic), but the level of normative expertise (the ability to make quantitative judgements) would vary greatly [25]. There is a risk that participants using distance elicitation, who have the intended expertise but not the skills to adequately formulate their opinion or even use the provided elicitation tool, do not complete the task. However, if they are assisted by a facilitator, they would be able to provide their opinion as probability distributions. This could lead to a possible bias in any aggregation of the individual distributions.

The aim of the study was to assess the practicality of a self-administered elicitation tool by exploring whether the elicited beliefs of the experts were influenced by the mode of elicitation (face-to-face with a facilitator vs. distance self-administered). Response rates among experts agreeing to participate, and time taken to complete the task, were also compared for the two modes. We hypothesised that, although potentially victim to a lower response rate, the distance approach would produce a higher number of complete responses, due to a larger potential sample than face-to-face elicitation.

The objectives of the study were to:

- develop a tool for the self-administration of an elicitation task;
- qualitatively compare probability distributions elicited from experts via face-to-face interviews with those elicited via email;
- compare feasibility of the two modes of administration in terms of trading off response rates and time taken to complete elicitation for access to more experts.

## Methods

I. **Pre-elicitation**

### Study design

The study was designed as a two arm parallel trial, with experts randomly assigned to:

- face-to-face elicitation;
- distance elicitation via email.

Regardless of the mode of elicitation, expert opinion was elicited using the same tool (see below for details).

### Choice of elicitation topic and experts

From previous experience [18], and the literature [2, 17], low response rates were anticipated [26]. Consequently, the elicitation was aimed at a group of health professionals that were numerous: general practitioners (GPs). A hypothetical decision problem based on drinking behaviours was chosen, inspired by an existing research stream [27], so that GPs would have knowledge in this area.

### Recruitment of the experts

GPs and GPs in training, based in the UK, were considered as appropriate experts for this study.

Primary care research networks and GP training centres in Devon, UK were contacted and invited to participate in the study. As this resulted in few responses, a snowballing approach [28] was used to increase the sample. Experts willing to participate were randomised to the two methods using a pre-defined randomisation list; participants in the face-to-face group were further contacted to schedule a meeting.

Recruitment continued until either: a) the target number of 12 participants was recruited to each group, or b) a period of 6 months allocated for recruitment ended. The target number of participants was based on recommendations regarding the necessary number of experts for a typical elicitation exercise [17, 25].

All recruited experts were provided with an information leaflet that included details on the study, an indication of what the elicitation session would entail (including the cognitive burden for the expert, time commitment and opportunity to learn more about representing uncertainty) and information on confidentiality measures (specifically that individual estimates would be anonymised in any public report).

II. **During the elicitation**

### The EXPLICIT elicitation tool

An elicitation tool was necessary for the elicitation sessions, which experts could use by themselves with some assistance (in face-to-face elicitation) or no assistance (in distance elicitation). Among the readily available elicitation tools, none was directly usable in this study, as they were generally intended for specific fields or covered only the encoding part of the elicitation. The tools, briefly described below, were used to help develop a bespoke elicitation tool. Devilee and Knol 2012 reviewed available elicitation packages [29]., and found limited support available in the tools for experts, including a lack of preparation of the expert for the elicitation task.

Tools to be used with a facilitator include the SHELF (SHeffield ELicitation Framework) tool [30], which uses R and provides visual representations based on summaries provided. Although it could be used for individual face-to-face elicitation, it is mainly intended for group elicitation. The Bias Elicitation in Evidence Synthesis (BEES) [31] tool is a graphical interface for bias elicitation [32], limited to the elicitation of 67% confidence intervals on a log scale. Elicitor [33, 34] and Expert CALIBRation (EXCALIBUR) [35] are specifically intended to be used in environmental risk assessment, and provide limited ways in which to specify distributions and, most importantly, require a great deal of specific knowledge to deploy and use.

More recently, tools have been developed for remote elicitation. The MATCH Uncertainty Elicitation Tool [36] was developed as a web-based interface for SHELF. Pibouleau et al. [22] describe another web-based interface for the hybrid encoding method, while others [19, 20] have reported using Excel-based questionnaires that were circulated by email. Sperber et al. [21] improved on this concept by integrating visual and quantitative feedback and a short introduction to the elicitation context.

The elicitation tool needed for this study had to fulfil several requirements:

- be able to be used by the expert in the absence of a facilitator;
- be used on different platforms;
- ensure the preparation of the expert for the elicitation session (i.e. ensure the expert is given the required information and training to complete the elicitation task);
- ensure that good elicitation practice is observed;
- control for potential risks of bias in the elicitation.

To fulfil these requirements, an Excel-based tool (**“EXPLICIT”, EXPert eLICItation Tool**) was developed. EXPLICIT was based on the Excel template used in a previous study [18], which allowed for visual feedback to the expert and real-time fitting of smooth parametric distributions to the histogram based on the expert’s summaries.

However, the previous tool was designed to be operated by a facilitator during the elicitation session. The role of the facilitator included explaining the context and instructing the expert on how to express his/her uncertainty, and ensure that the summaries the expert gave were coherent and had face validity. These steps were subsequently implemented in the EXPLICIT tool.

EXPLICIT prepares the expert for the elicitation task, displays the questions, records the answers, provides visual feedback to the expert, and checks the consistency of the estimates.

**Fig. 1:**
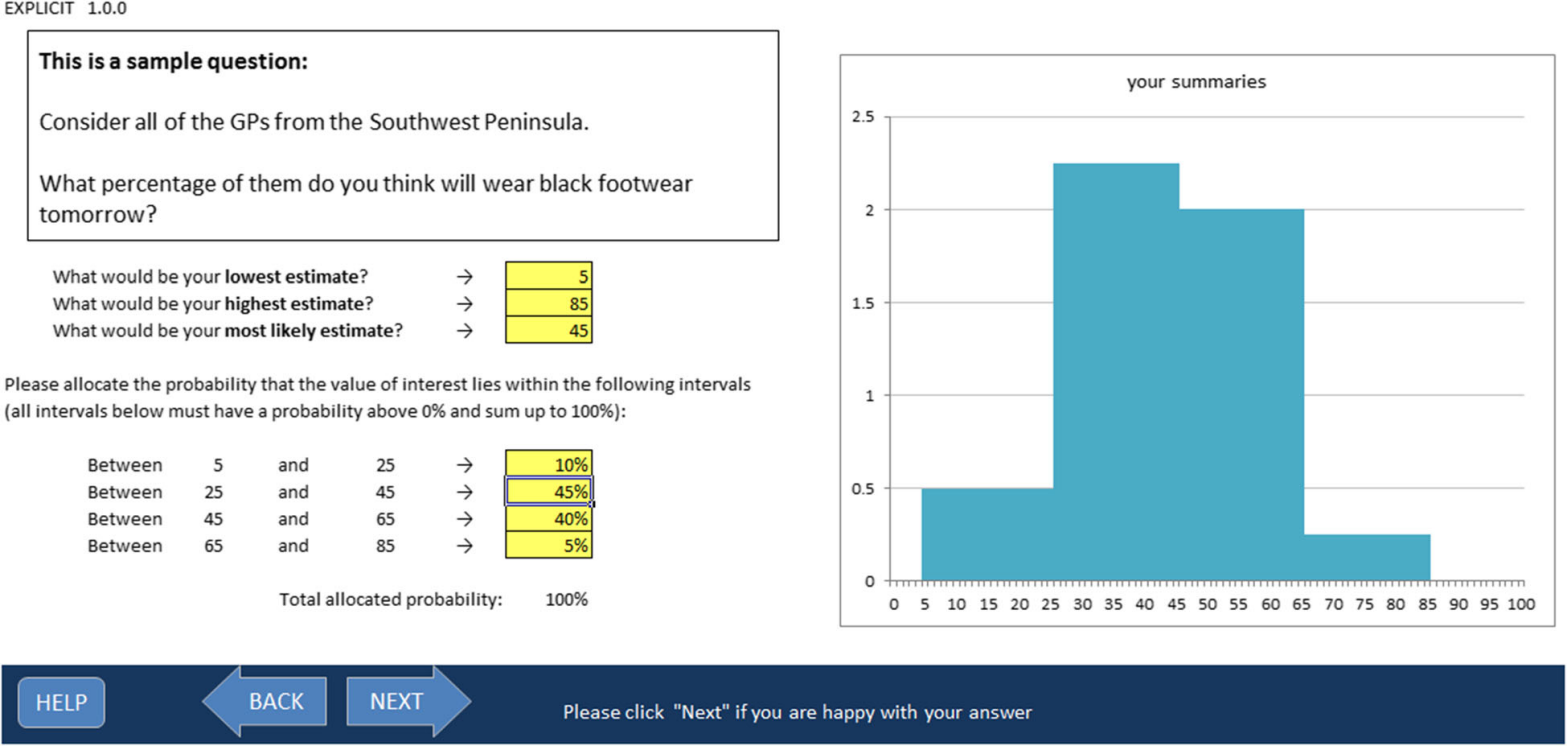
EXPLICIT training question. The expert practices using the tool to provide his estimate in response to a mock question

EXPLICIT has three sections: introduction, training and questionnaire. The introductory section contains general information about the tool and the study, and an electronic consent form. The training section contains:

- short introduction to probability assessment including:
- explanation of probability, proportions, uncertainty, probability distributions;
- examples of probability distributions given in response to a fictional question;
- instructions on how to provide expert opinion using EXPLICIT and an opportunity to complete an **example question** (see Fig. 1);
- summary screen, where the expert is made aware of common potential biases and is encouraged to control for them.

While many encoding methods can be implemented in EXPLICIT, the method implemented for this study was the hybrid method (previously described as “four complementary intervals” [19]). It was chosen as it allows for intuitive understanding of what the distribution represents since it is based on the probability density function [37]. The hybrid method is more flexible than fixed interval methods (like the histogram technique), so allows the recording of distributions for both proportions and scalar quantities. A review of practice [38] showed that it has often been applied in HTA [19, 22], experts have expressed a preference for the hybrid method compared to the histogram method [18].

In the hybrid method, three estimates of the quantity of interest are initially elicited: the lower limit “L”, the upper limit “H” and the mode “M”; the intervals M-L and H-M are each divided in half and the expert is asked to allocate the probability of the event in question to each of the resulting four intervals [19] (see Fig. 1).

The questionnaire section collects elicited summaries, verifies their consistency, encodes them as a histogram and ensures that the expert is satisfied with the representation of their estimate (i.e. face validity). Consistency verification includes checking that: 100% of the probability is allocated to the histogram, “L < M < H”, the expert does not provide a uniform distribution (by reminding the expert that, by definition, M suggests more probability allocated towards it) and, the extreme values do not cover the entire range, suggesting complete uncertainty.

A smooth parametric distribution can be fitted to the histogram, provided the EasyFitXL plugin [39] is installed on the computer on which EXPLICIT is running.

EXPLICIT records the time taken to complete the training section and the questionnaire section.

EXPLICIT was designed with wide platform compatibility and can be run as a software application on any system that has Microsoft Excel 2003 or later installed; or under Libre Office Calc (with minor compatibility issues).

#### Questionnaire

The following elements were included in the questionnaire:

1. **general questions** about the expert: gender, age group, previous experience with elicitation, level of knowledge of statistics, number of years working as a GP, number of relevant cases seen in an average month (“Approximately how many patients with (known or suspected) alcohol-related health problems (acute or chronic) do you see in an average month?”);
2. **seed question**; this output was not used in this study but was used for a related study reported elsewhere [40]:

“Think of the entire adult population (ages 16 and over) in Great Britain. What percentage of them do you think drank alcohol in the last week?”

3. **main question:** “According to Mann et al. (2003) [41], around 20% of heavy drinkers develop fatty liver, or steatosis. This condition can lead to death if the drinking behaviour continues, but it can also be reversed if alcohol consumption is *stopped or significantly reduced*. As part of the patient’s visit, GPs have the opportunity to deliver brief interventions lasting 5–10 min (Angus et al. 2014) [42] to patients at risk.

Now, consider 100 randomly selected patients in Great Britain that have been diagnosed with hepatic steatosis (fatty liver) due to consumption of alcohol.

How many of them would stop OR significantly reduce the amount they drink when finding out about their alcohol-related health problems WITHOUT additional interventions beyond GP’s advice?”

4. **a visual analogue scale** asking the experts to quantify their certainty about the answer provided to the main question; this question was relevant only for a related study [40].

III. ** Post-elicitation**

The characteristics of individual estimates, response rates and time to complete the elicitation session were compared between the two arms. Individual distributions obtained from each arm were arithmetically averaged, and beta distributions fitted using the EasyFit XL application [39]. The resulting distributions were compared qualitatively.

### Piloting

Several piloting sessions were run internally within the University of Exeter Medical School to ensure consistent operation of the tool. It was also validated during the first face-to-face session, to ensure that the content was easy to understand and that the expert could complete the questionnaire consistently.

### Ethics

Ethics approval was obtained from the University of Exeter Medical School Research Ethics Committee.

## Results

### Recruiting

**Fig. 2:**
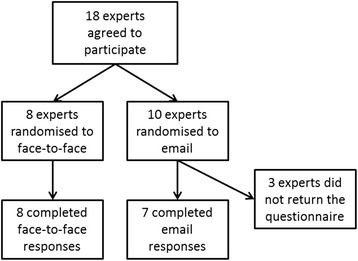
Flow diagram of experts’ participation in the study

Recruitment lasted from April to November 2014, and 18 GPs agreed to take part in the study. At the end of the data collection period, eight completed questionnaires were available from the face-to-face group and seven from the distance elicitation group (see Fig. 2), therefore completion rate from randomisation was 100% for face-to-face and 70% for email. A response rate taking into account all potential participants targeted by the invitation could not be calculated, as the recruiting approach, which included snowballing, did not allow for calculation of the number of experts receiving the request to participate.

While the outputs of the face-to-face sessions were immediately available to the researcher, it took between three to eleven email or telephone “reminders” over the course of 6 weeks to obtain the email responses.

Of the three experts that did not complete the emailed questionnaire, one reported difficulty providing probabilistic judgements, another dropped out due to maternity leave, and nothing was received from the third expert, despite multiple attempts to contact him.

### Consent to participate and to publish

At the beginning of each session, consent to participate and to publish were obtained from the participating experts.

### Sample structure

Table 1 presents the general characteristics of the two groups of experts.

**Table 1:** Characteristics of the participating experts

Characteristic	Face-to-face elicitation group (total = 8)	Email elicitation group (total = 7)
*Gender (count)*		
Female	3	3
Male	5	4
*Age group (count)*		
Under 30	2	1
31–35	3	1
36–40	1	3
41–45	2	1
46–50	-	1
Over 50	-	-
*Previous elicitation experience (count)*		
Yes	-	-
No	7	6
Not sure	1	1
*Familiarity with statistics (count)*		
Fair	4	7
Good	3	-
Very good	1	-
*Number of years of experience as a doctor (mean, standard deviation)*	9 (5)	10 (6)
*Number of relevant patients seen per month* ^a^ *(mean, standard deviation)*	24 (21)	21 (15)
*Time to complete the questionnaire in minutes:seconds (mean, standard deviation)*		
Total	10:26 (4:54)	13:30 (2:59)
Training	05:43 (4:09)	07:01 (2:10)
Actual questionnaire	04:43 (1:35)	06:29 (1:46)

Note: ^a^Refers to the average number of patients with known or suspected alcohol-related health problems

The face-to-face and email groups were similar regarding gender composition, previous contact with elicitation, average number of years’ experience as a GP and average number of patients, with known or suspected alcohol-related health problems, seen per month. More experts in the face-to-face group assessed their familiarity with statistics as “good” or “very good”, compared with the email group, where all experts evaluated their knowledge of statistics as “fair”.

### Amendments to the tool

As a result of the discussion during the first face-to-face elicitation session, a number of restrictions were implemented in the tool to prevent the expert giving illogical answers.

### Time to complete

The average time to complete the questionnaire was shorter in the face-to-face arm than the email arm (10 min 26 s vs 13 min 30 s, *p* = 0.1). When excluding the pilot session, time to complete the face-to-face session was even shorter compared to the email arm (8 min and 50 s vs 13 min 30 s, *p* = 0.008). Experts in the email arm spent longer in both components of the elicitation tool (see Table 1), compared to the face-to-face arm.

### Probability distributions

**Fig. 3:**
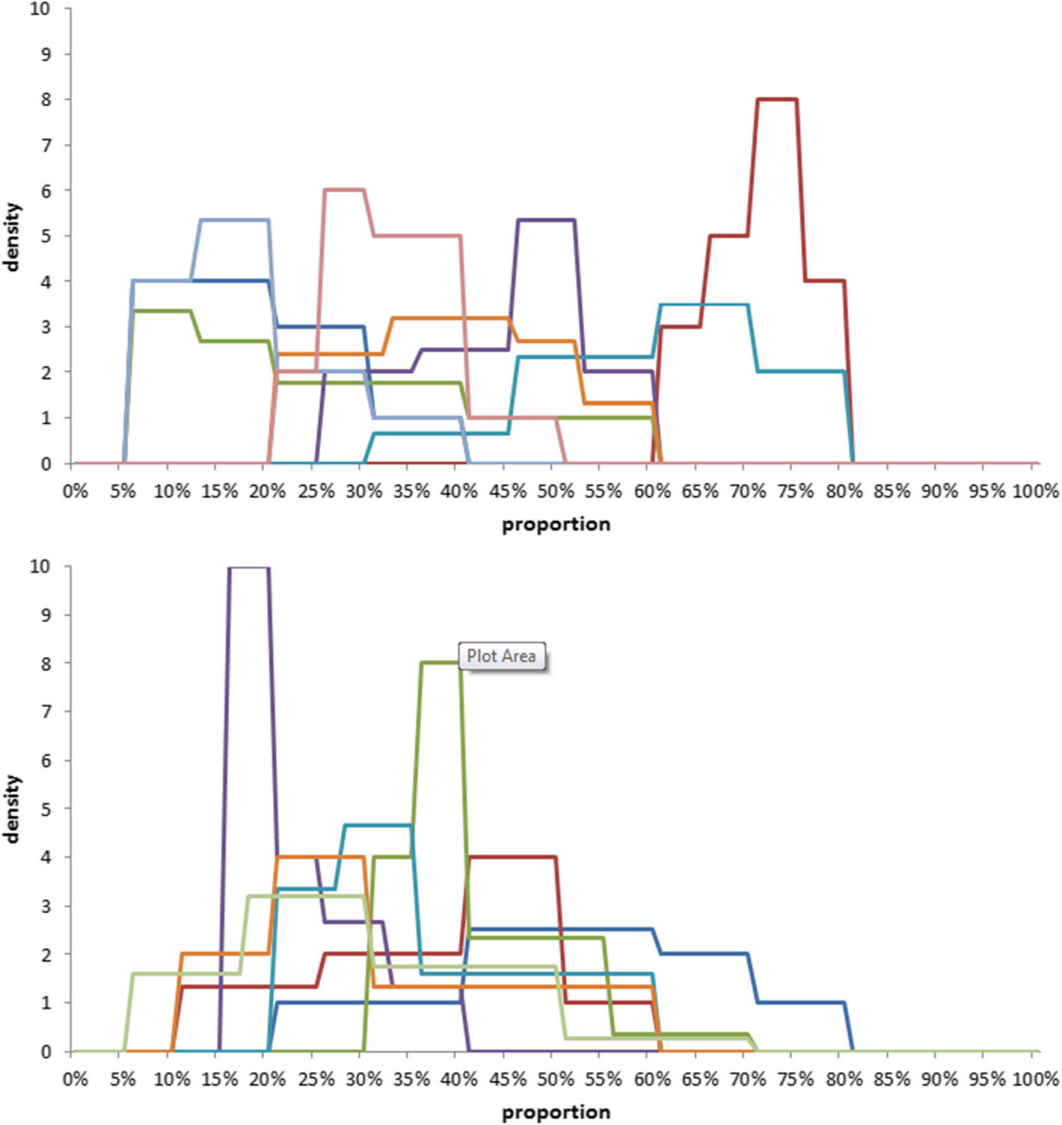
Individual probability distributions obtained from the experts in the: top – face-to-face arm; bottom – email arm

The individual histograms based on the experts’ summaries from the face-to-face and distance arms are represented in Fig. 3.

The means of elicited modes were similar for the two arms: 0.39 for the face-to-face arm, and 0.37 for the email arm. The email-elicited modes were less variable (min = 0.25, max = 0.6) compared to the face-to-face arm (min = 0.2, max = 0.7).

On average, distributions elicited by email were more uncertain, having wider ranges. The mean range (H-L) of all of the elicitations was 0.38 for the face-to-face arm and 0.47 for email arm.

### Combined distributions

**Fig. 4:**
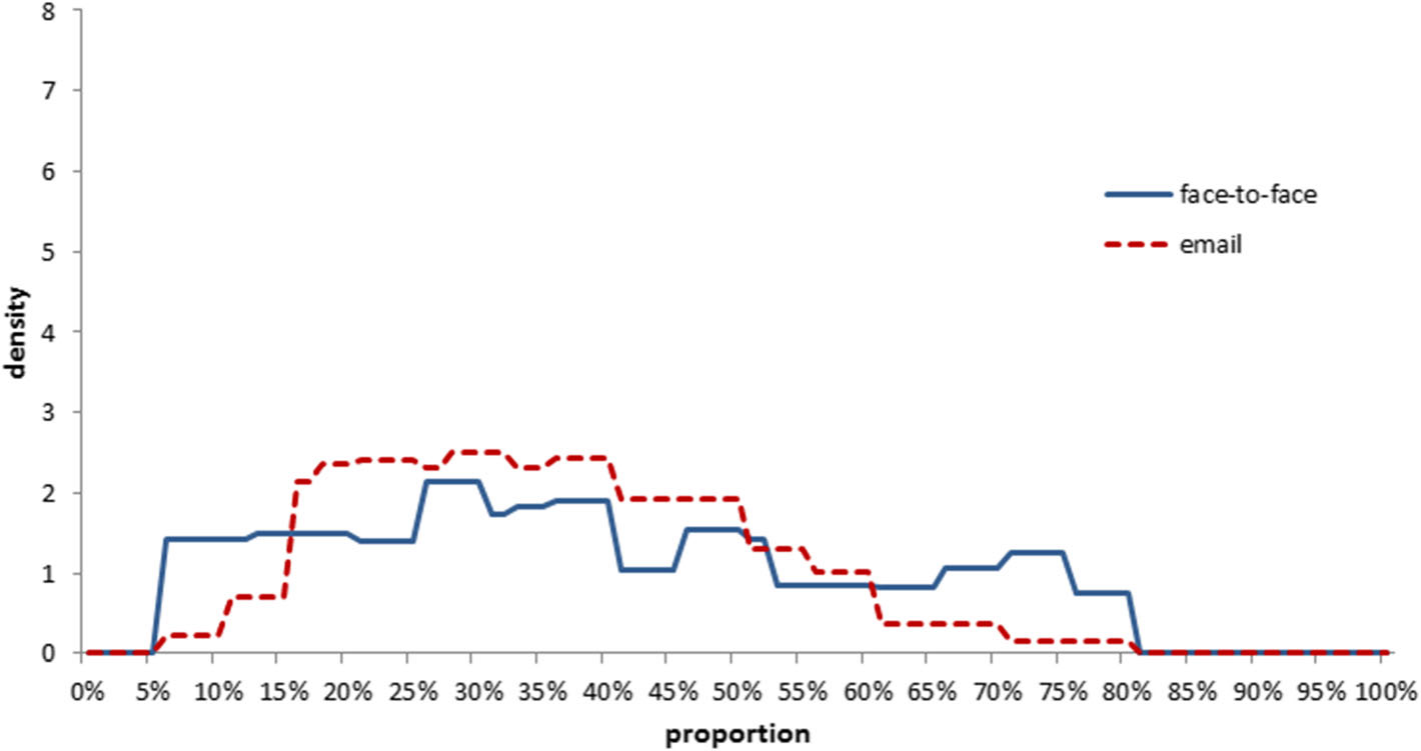
Averaged histograms for the two arms

The resulting combined distributions for each arm are presented in Fig. 4. The mean of the face-to-face distribution was 0.39 (standard deviation 0.2) and the mean for the email distribution was 0.36 (standard deviation 0.15). As email-elicited distributions were closer to each other, the combined distribution suggests a higher certainty about the true value.

**Fig. 5:**
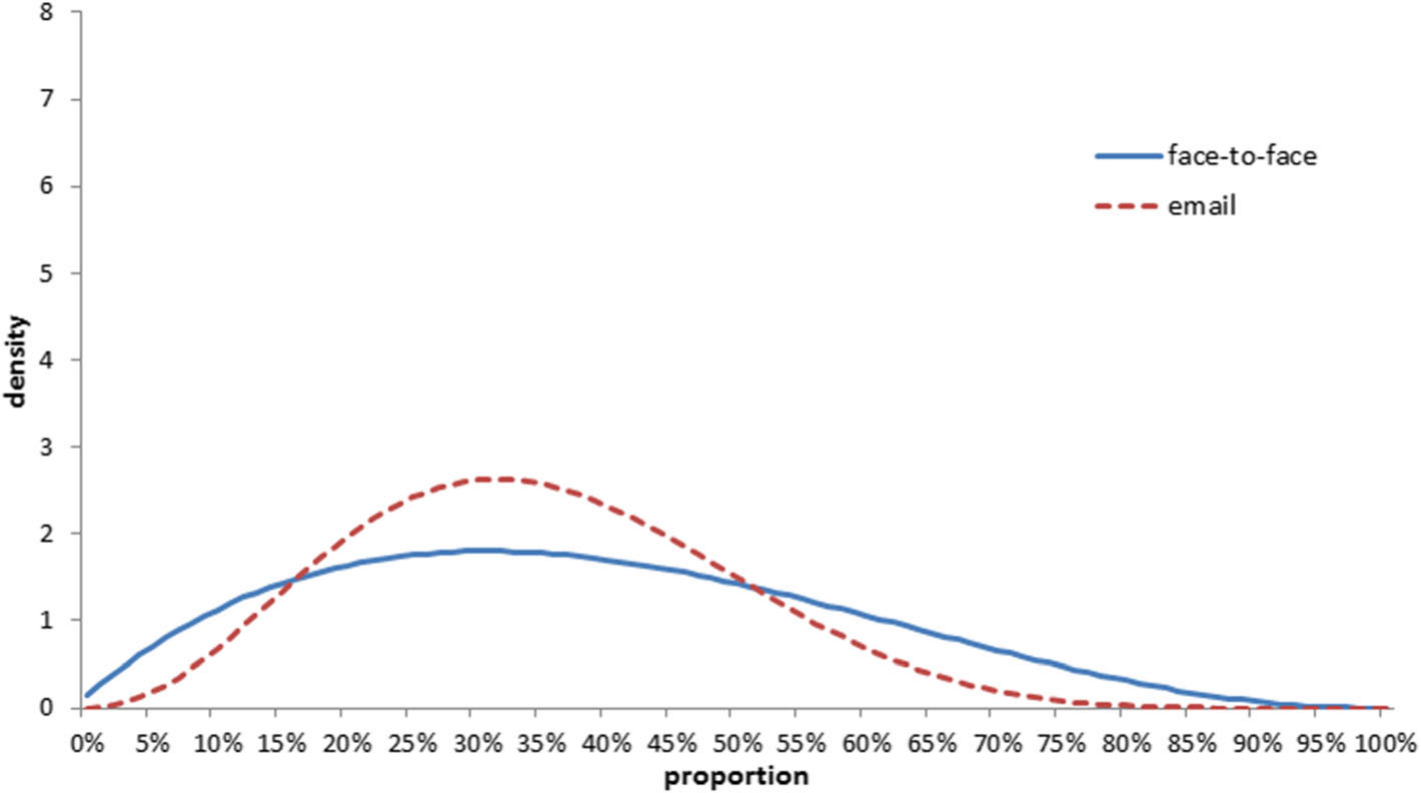
Fitted beta distributions to the combined distributions of each arm

Figure 5 shows the combined distributions fitted with beta distributions.

Face validity was assessed by asking the experts to confirm whether the visual representation offered by EXPLICIT was an accurate representation of their belief.

## Discussion

Using an Excel-based elicitation tool, probability distributions were obtained with the stated intention of informing a hypothetical decision problem.

Recruitment was very slow, taking several months. Even among the experts who explicitly agreed to participate, some dropped out of the study before completing the elicitation session. Recruiting through snowballing was more successful than approaching potential participants through the official research and training organisations. Some participants commented that this might be because official channels were saturated with various requests and many GPs would ignore messages not directly related to their activity. Another possible barrier to recruitment in the early stages was the lack of incentives for the participants. Towards the end of the recruiting period, a new invitation to participate was circulated where the focus was changed from the methods explored to the drinking behaviours topic, which recognised the potential for learning more about representing uncertainty. This might have contributed to more participants joining the study towards the end. Nonetheless, our approach to sampling, as well as the relatively small number of respondents, limit the generalisability of the findings.

It took longer to obtain data from the email participants than it took to schedule meetings in the face-to-face arm contrary to the hypothesis that email would result in more responses more quickly. A possible explanation is that the email participants were less motivated to engage in and complete the elicitation, because the objective and its deadline were perceived as very abstract. In contrast, by scheduling a time for the elicitation session, the objective was clearer for the participants in the face-to-face group. These findings seem to confirm the evidence collected previously [18] regarding practical issues with the duration of expert recruitment.

Our findings show that it took longer for experts to complete the elicitation session by email than in face-to-face sessions. Participants in the email arm spent more time on both the training part and the questionnaire sessions than the face-to-face arm. The differences were, however, only a few minutes. Facilitation may have helped by increasing the confidence of the participants in the face-to-face arm. All of the sessions (whether face-to-face or distance) were completed in a reasonable time. One of the possible reasons for the good rate of successful elicitation sessions was the elicitation tool, which achieved a good balance of instructions and tasks, while also managing the cognitive burden of the exercise.

The individual distributions elicited in the face-to-face and distance arms were comparable. The distributions elicited by email were more uncertain, with wider intervals; however the means were more grouped together than in the face-to-face arm. It is unclear if this was an effect of different approaches, or a difference in the participating experts, for instance, the difference in familiarity with statistics as rated by the experts. When combining individual distributions for each arm, the aggregated distributions were still comparable. These findings were similar to existing studies exploring self-administered elicitation [23]. We did not perform further quantitative comparisons of the distributions; however, given the context of a decision model used for HTA purposes, the ultimate practical comparison should have been a comparison of model outputs when the model is informed by each of the distributions.

Because of the computer-assisted nature of the task, it was possible, in theory, to favour the recruitment of experts who had better computer literacy, but not necessarily “better” expertise. However, to compensate the impact of selection bias, the elicitation question addressed a relatively common phenomenon that most GPs were believed to be able to express in a quantitative way. A ‘Think Aloud’ analysis [43] would have provided deeper insight into the interaction with the tool and respondents’ choices; this was not done as part of the study, but such a protocol is been prepared for the future development of EXPLICIT.

Another potential source of bias is the recording of inconsistent distributions. This is a risk especially with distance elicitation. EXPLICIT limited the possibility of this situation occurring by doing several real-time consistency checks to the summaries introduced by the experts. However, EXPLICIT does not prevent the expert from building a highly skewed distribution, even when common sense might suggest a normal distribution. Arguably, for facilitated sessions, such situations could be discussed between the facilitator and the experts, leading to a more consistent distribution, but this was not possible for email elicitations.

Finally, distance elicitation does not have to be completely devoid of facilitation. This study attempted to isolate the impact of facilitation by only providing it in the distance arm through the elicitation tool. It would be possible, however, to provide facilitation by phone or in a video-conference to an expert. Scheduling phone calls to take the expert through the elicitation exercise might also increase the chances of obtaining the responses in a timely manner.

## Conclusions

Outcomes were comparable between the face-to-face and email elicitation groups. Recruiting is, however, a key issue and the potential experts must be vigorously pursued, especially in a time-limited HTA context, regardless of whether the session is conducted face-to-face or remotely; this is an even more critical issue if the experts are scarce. Remote self-administered elicitation can be a viable approach when experts are not immediately available for a face-to-face session and EXPLICIT can be used successfully to achieve this, possibly at the expense of poorer response rates and additional time needed for the elicitation sessions.

## Abbreviations

BEES: Bias Elicitation in Evidence Synthesis
EXCALIBUR: Expert CALIBRation
EXPLICIT: EXPert eLICItation Tool
GP: General practitioner
SHELF: SHeffield ELicitation Framework
UK: United Kingdom
